# ASO Author Reflections: Multivisceral Versus Tumor-Only Resection in Retroperitoneal Liposarcoma

**DOI:** 10.1245/s10434-024-15090-6

**Published:** 2024-02-26

**Authors:** Amblessed Onuma, Leva Gorji, Joal D. Beane

**Affiliations:** 1 Department of Surgical Oncology, The University of Texas MD Anderson Cancer Center, Houston, TX USA; 2 Department of Surgery, Kettering Health Dayton, Dayton, OH USA; 3 Department of Surgery, The Ohio State University Wexner Medical Center and James Cancer Hospital, Columbus, OH USA

## Past

Surgical resection of retroperitoneal liposarcoma with grossly negative margins is a good prognostic indicator in the surgical management of this disease.^1,2^ Two operative strategies (tumor-only and multivisceral resection) have evolved through the years. Multivisceral resection involves excision of the tumor in addition to adjacent organ-abutting of the tumor to ensure adequate negative margins, whereas tumor-only resection involves resection of only gross disease. Prior studies have not shown any clear difference between the two operative techniques.^3^

## Present

This retrospective study compared the two operative strategies using the win ratio, a novel approach that analyzes the composite end points of two competing approaches.^4^ The advantage of this method is that it prioritizes end points in a hierarchical order to achieve a set benefit. The authors chose 30 day mortality, 90 day mortality, overall survival, and severe complication (defined as readmission within 30 days after surgery) as their end points prioritized in that order. The study found no difference in overall win ratio between the patients who underwent multivisceral resection and those who underwent tumor-only resection in the matched cohort. In subgroup analysis, the patients 72–90 years of age had lower odds of winning if they underwent multivisceral resection instead of tumor-only resection. The patients who had no adjacent tissue involvement of their tumor and underwent multivisceral resection experienced lower odds of winning than those who underwent tumor-only resection.

## Future

Due to the retrospective nature of this study, the authors were unable to ascertain the justification for organ resection or the number of organs resected. The results suggest that for a certain age group, multivisceral resection increases morbidity without providing an overall benefit. These findings highlight the need to further optimize patient selection and the appropriate operative approach in the management of retroperitoneal liposarcoma. Future directions include a large-scale, prospective study to improve understanding of the surgical management of this disease.
